# Uptake and Utilization of the Management of Anticoagulation in the Periprocedural Period App: Longitudinal Analysis

**DOI:** 10.2196/11090

**Published:** 2018-12-21

**Authors:** Alex C Spyropoulos, Anne Myrka, Darren M Triller, Stephen Ragan, Collin York, Jaz-Michael King, Ti-Kuang Lee

**Affiliations:** 1 Donald and Barbara Zucker School of Medicine at Hofstra/Northwell New York, NY United States; 2 IPRO Albany, NY United States; 3 WellScriptEd Consulting Inc Delmar, NY United States

**Keywords:** adverse drug event, anticoagulant, app, mobile phone, periprocedural, warfarin

## Abstract

**Background:**

Anticoagulants are major contributors to preventable adverse drug events, and their optimal management in the periprocedural period is particularly challenging. Traditional methods of disseminating clinical guidelines and tools cannot keep pace with the rapid expansion of available therapeutic agents, approved indications for use, and published medical evidence, so a mobile app, Management of Anticoagulation in the Periprocedural Period (MAPPP), was developed and disseminated to provide clinicians with guidance that reflects the most current medical evidence.

**Objective:**

The objective of this study was to assess the global, national, and state-level acquisition of a mobile app since its initial release and characterize individual episodes of use based on drug selection, procedural bleeding risk, and patient thromboembolic risk.

**Methods:**

Data were extracted from a mobile app usage tracker (Google Analytics) to characterize new users and completed episodes temporally (by calendar quarter) and geographically (globally, nationally, and in the targeted US state of New York) for the period between April 1, 2016 and September 30, 2017.

**Results:**

The app was acquired by 2866 new users in the measurement period, and the users completed nearly 10,000 individual episodes of use. Acquisition and utilization spanned 51 countries globally, predominantly in the United States and particularly in New York State. Warfarin and rivaroxaban were the most frequently selected drugs, and completed episodes most frequently included the selection of high bleeding risk (4888/9963, 49.06%) and high thromboembolic risk categories (4500/9963, 45.17%).

**Conclusions:**

The MAPPP app is a successful means of disseminating current guidance on periprocedural anticoagulant use, as indicated by broad global uptake and upward trends in utilization. Limitations in access to provider and patient-specific data preclude objective evaluation of the clinical impact of the app. An ongoing study incorporating app logic into electronic health record systems at participant health systems will provide a more definitive evaluation of the clinical impact of the app logic.

## Introduction

Anticoagulants are highly prescribed medications that have been identified as major contributors to adverse drug events (ADEs), of which bleeding is the most common and dangerous. Anticoagulants have repeatedly been identified among the drugs most frequently associated with emergency department (ED) visits, and national estimates indicate that the rates of such events are increasing [1-4]. While warfarin had been the only available oral anticoagulant for decades, 5 new direct oral anticoagulants have come to market in the United States since 2010, paralleled by an increase in outpatient anticoagulant use [5]. The proportion of ED visits due to anticoagulants has increased by 57% in that period, and anticoagulants now account for nearly 18% of all ADE-associated ED visits in the United States [1-4,6]. Because many serious ADEs are thought to be preventable through improvements in care delivery, anticoagulants are identified as a specific target for health system improvement in the US Department of Health and Human Services’ National Action Plan for Adverse Drug Event Prevention [7-16]. Quality Innovation Network-Quality Improvement Organizations (QIN-QIOs) have also been contracted by the Centers for Medicare & Medicaid Services (CMS) to work with providers, Medicare beneficiaries, and other stakeholders to improve the quality of care for patients who are prescribed anticoagulants [17].

For patients utilizing chronic oral anticoagulation, medical management in the time period leading up to and following an invasive medical procedure or surgery (ie, the periprocedural period) is particularly challenging. Such patients are exposed to an increased risk of serious or life-threatening bleeding when clinically relevant anticoagulation is present in the absence of complete hemostasis, and conversely, they are at increased risk of thrombosis when the degree of anticoagulation present is insufficient to address underlying thromboembolic risk (ie, when an anticoagulant is held for surgery). Under such circumstances, clinicians must balance the risk of bleeding associated with the procedure with that of the underlying thromboembolic risk of condition(s) that has prompted the need for anticoagulation. With approximately 10%-15% of chronically anticoagulated patients requiring anticoagulant interruption for an invasive procedure each year, experts have endeavored to create guidance for optimal periprocedural management, addressing surgical risk, thromboembolic risk, and the pharmacokinetic profiles of the individual anticoagulants [18-23]. However, the rapid expansion of available agents, approved indications for use, and related medical evidence serve to minimize the utility of traditional published guidelines, which are revised infrequently and are not reflective of the most current practices [24]. Similarly, it can be difficult to recall or update clinical tools disseminated in hard copy or pdf formats, and neither supports passive data collection on utilization or impact on patient care.

To increase clinician access to the most current expert guidance on periprocedural anticoagulation management, the New York State Anticoagulation Coalition (NYSACC) led the development and dissemination of the Management of Anticoagulation in the Periprocedural Period mobile app (MAPPP; see Multimedia Appendix 1) [25]. This paper describes the creation of the app and its adoption and utilization globally, in the United States, and in the state of New York. It also characterizes episodes of use by drug, procedural bleeding risk, and patient thromboembolic risk.

## Methods

### Clinical Tool Development

The NYSACC was created in 2012 by IPRO, the CMS-designated QIO for New York State, to advance the drug safety priorities identified in the CMS QIN-QIO 10th Statement of Work [26]. The NYSACC’s multidisciplinary membership consists of more than 150 representatives from clinical practice, academia, industry, and advocacy organizations with interest in anticoagulation management quality. The NYSACC identified inadequate availability of tools for periprocedural management as a barrier to quality anticoagulant management that warranted action.

In the spring of 2013, the group developed and disseminated a novel clinical tool, the MAPPP Tool. This tool, disseminated in hard copy and pdf formats, utilized a 3×3 matrix to help clinicians simultaneously categorize underlying thromboembolic risk (high, moderate, or low) and procedural bleeding risk (high, low, or minimal) and provided evidence-based guidance on (1) whether the anticoagulant should be interrupted; (2) timing for preprocedural discontinuation of anticoagulant (if necessary); (3) whether “bridging” with heparin products is warranted, with details of timing and laboratory monitoring; and (4) timing and dosing of re-initiation of anticoagulant in the postprocedural period. The tool included guidance for all oral anticoagulants available for use in the United States at the time of release, including apixaban, dabigatran, edoxaban, rivaroxaban, and warfarin.

### App Development

Recognizing the barriers to disseminating and updating the MAPPP in its original form, and appreciating the limited number of mutually exclusive steps involved in navigating the tool, it was determined that the structure of the MAPPP lent itself to the creation of a mobile app that would potentially allow for broader dissemination, remote updates to reflect most current knowledge, and the collection of data on downloads and utilization. The MAPPP app design team was convened, which included clinical content experts and IPRO app developers, with the goal of creating an app that would provide a high-value proposition for the clinical user (evidence-based, accurate, and quick results) with simplicity of design (few pages). Construction of a wireframe and subsequent user interface sample screenshots using MAPPP app logic were designed to iteratively develop the app through use cases, preliminary usability testing, and clinical content expert feedback. The resulting prototype was refined into the final product via iterative cycles of clinical user testing.

The MAPPP app was developed using the open source framework Cordova (Apache Software Foundation, Wakefield, MA), which utilizes HTML, CSS, and JS to develop apps across multiple platforms from a single code base. The MAPPP app was made available through both the iOS App Store and Google Play; it functions with Web browsers that support modern Web technologies. The app was released in April 2016 and, based on user feedback, underwent minor modifications in May 2016 to enhance the disclaimers, improve the visibility of the button linking to more information on selection options, and expand flexibility of use through the addition of a “Back” button. While no major changes have been made to the clinical content of the app to date, a process is in place to support app updates as necessary based on the most current evidence-based recommendations in the field of perioperative antithrombotic therapy.

### Analysis of App Uptake and Utilization

Data were extracted from Google Analytics (including iOS, Android, and Web browser data) to characterize new users and completed episodes temporally (by calendar quarter) and geographically (globally, United States, and the targeted US state of New York) for the period between April 1, 2016 and September 30, 2017. A completed episode was defined by the project team as a user interaction that resulted in the app presenting a recommendation page based on sequential selection of an anticoagulant drug, assignment of bleeding risk category for a procedure, and indication of underlying thromboembolic risk of the patient [21]. Additional analysis of completed episodes was performed to characterize utilization by individual drug, procedural bleeding risk category, and underlying thromboembolic category.

## Results

**Figure 1:**
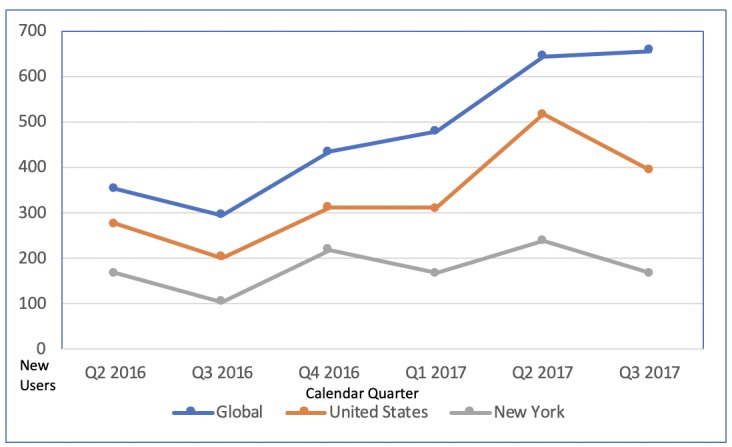
New users of the Management of Anticoagulation in the Periprocedural Period App by calendar quarter (Q) as defined by Google Analytics.

**Figure 2:**
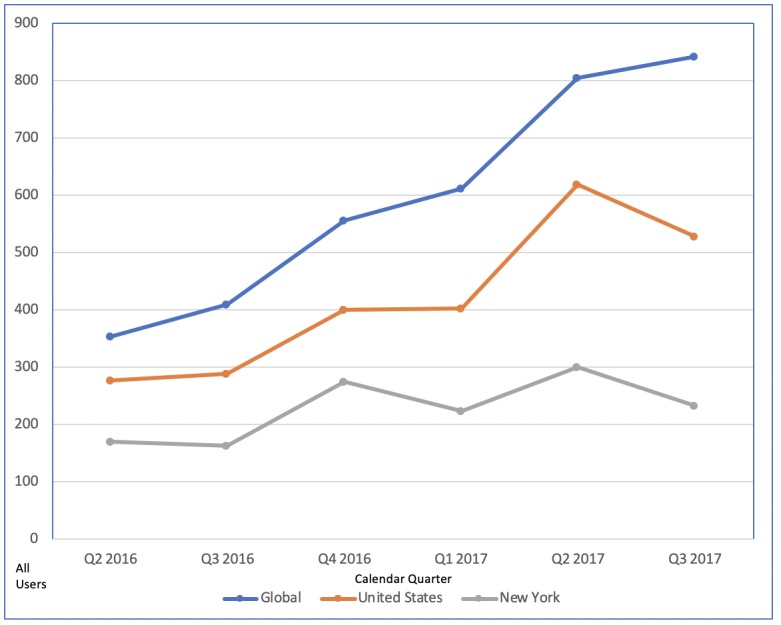
All users of the Management of Anticoagulation in the Periprocedural Period App by calendar quarter (Q) as defined by Google Analytics.

There were 353 new users of the app globally in its first full calendar quarter of use, primarily in the United States (276/353, 78.18%; Figure 1) and in the project’s home state of New York (168/353, 47.59%). App acquisition increased through Q3 2017, accruing 2866 total global new users, dominated by use in the United States (2013/2866, 70.24%), particularly in New York (1067/2866, 37.23%). The app was downloaded and used for at least one episode in 51 different countries during the measurement period. Among US states, California (85) and New Mexico (69) had the second and third greatest numbers of new users. The quarterly total of all users (ie, the sum of new and returning users) trended upwards in like manner (Figure 2).

Users completed nearly 10,000 episodes during the study period, exceeding 2000 episodes in each of the most recent calendar quarters (Table 1). Overall, utilization was highest in the United States (6748/9963, 67.73%), particularly in New York (3618/9963, 36.31%). Globally, episodes were spread approximately evenly between new users (4571/9963, 45.88%) and returning users (5392/9963, 54.12%).

Among the completed episodes, the most commonly selected medications were warfarin (4074/9963, 40.89%), followed by rivaroxaban (2347/9963, 23.56%; Table 2). Each completed episode required the selection of a drug, a procedural bleeding risk category, and an underlying thromboembolic risk category, thus, allowing for 45 possible drug-risk-risk combinations. Of these possible combinations, those indicating high bleeding risk (4888/9963, 49.06%, episodes) and high thromboembolic risk (4500/9963, 45.17%, episodes) were the most commonly selected. Warfarin at 13.29% (1324/9963) and rivaroxaban at 6.52% (650/9963) were the agents most frequently associated with high bleeding risk–high thromboembolic risk episodes.

More in-depth analysis of trends among drug selections and risk categorizations of completed episodes would require evaluation of drug utilization trends among individual nations, facility-level data (eg, procedures performed, patients characteristics), user profiles (eg, medical residents vs experienced clinicians), and other variables that were not available for the current analysis, so no such analysis was attempted.

**Table 1:** Completed episodes by calendar quarter.

Geography		Quarter 2 2016 (n=1083), n (%)	Quarter 3 2016 (n=1064), n (%)	Quarter 4 2016 (n=1718), n (%)	Quarter 1 2017 (n=1766), n (%)	Quarter 2 2017 (n=2071), n (%)	Quarter 3 2017 (n=2261), n (%)	Total to date (N=9963), n (%)
**Global**								
	New users	567 (52.35)	426 (40.04)	743 (43.25)	813 (46.04)	937 (45.24)	1085 (47.99)	4571 (45.88)
	Returning users	516 (47.65)	638 (59.96)	975 (56.75)	953 (53.96)	1134 (54.76)	1176 (52.01)	5392 (54.12)
**United States**								
	Total users	779 (71.93)	760 (71.43)	1169 (68.04)	1018 (57.64)	1588 (76.68)	1434 (63.42)	6748 (67.73)
	New users	432 (39.89)	279 (26.22)	466 (27.12)	476 (26.95)	721 (34.81)	612 (27.07)	2986 (29.97)
	Returning users	347 (32.04)	481 (45.21)	703 (40.92)	542 (30.69)	867 (41.86)	822 (36.36)	3762 (37.76)
**New York**								
	Total users	525 (48.48)	383 (36)	759 (44.18)	543 (30.75)	712 (34.38)	696 (30.78)	3618 (36.31)
	New users	255 (23.55)	140 (13.16)	297 (17.29)	261 (14.78)	302 (14.58)	262 (11.59)	1517 (15.23)
	Returning users	270 (24.93)	243 (22.84)	462 (26.89)	282 (15.97)	410 (19.8)	434 (19.2)	2101 (21.09)

**Table 2:** Details of completed episodes of use (N=9963).

Bleeding risk and thromboembolic risk		Apixaban, n (%)	Dabigatran, n (%)	Edoxaban, n (%)	Rivaroxaban, n (%)	Warfarin, n (%)	Row values, n (%)
**High**							
	High	526 (5.28)	368 (3.69)	132 (1.32)	650 (6.52)	1324 (13.29)	3000 (30.11)
	Moderate	215 (2.16)	169 (1.7)	35 (0.35)	319 (3.2)	560 (5.62)	1298 (13.03)
	Low	118 (1.18)	72 (0.72)	25 (0.25)	137 (1.38)	238 (2.39)	590 (5.92)
**Low**							
	High	189 (1.9)	99 (0.99)	29 (0.29)	256 (2.57)	430 (4.32)	1003 (10.07)
	Moderate	327 (3.28)	234 (2.35)	52 (0.52)	435 (4.37)	615 (6.17)	1663 (16.69)
	Low	200 (2.01)	84 (0.84)	21 (0.21)	151 (1.52)	343 (3.44)	799 (8.02)
**Minimal**							
	High	100 (1)	55 (0.55)	30 (0.3)	124 (1.24)	188 (1.89)	497 (4.99)
	Moderate	80 (0.8)	69 (0.69)	21 (0.21)	115 (1.15)	174 (1.75)	459 (4.61)
	Low	148 (1.49)	91 (0.91)	53 (0.53)	160 (1.61)	202 (2.03)	654 (6.56)
Total		1903 (19.10)	1241 (12.46)	398 (3.99)	2347 (23.56)	4074 (40.89)	9963 (100)

## Discussion

### Principal Findings

The MAPPP app was developed and disseminated to address a pressing public health need (ie, the rate of ADEs associated with anticoagulant use). The technology was perceived to have specific advantages over traditional distribution of clinical tools as hard copies or pdf files, including ease of access when providing direct patient care and the ability to reach an expanded recipient audience, update content, and passively collect data. This analysis of the initial uptake and utilization of the MAPPP app supports the premise that such apps can not only be a useful means of disseminating clinical tools but also help identify relevant weaknesses and opportunities for improvement.

The app demonstrated the ability to reach a broad audience of recipients within not only the state of New York (the target geography of the CMS-funded quality improvement project) but also across the United States and in other countries. Despite the absence of any formal dissemination plan beyond an initial launch press release on April 29, 2016, direct provider interactions in New York, and sharing by members of the NYACC, the app has continued to generate new users and a growing number of completed user episodes. Evaluation of utilization data also suggests that the app is frequently used to access guidance for patients undergoing procedures with the highest bleeding and thromboembolic risk. While no changes were made to the clinical content of the app during the period of this evaluation, several formatting changes were made successfully, demonstrating the ability to update information to providers without having to remove outdated hard copies or pdf files. Finally, the analysis was performed using data passively collected by Google Analytics, which is impossible with hard copy tools or pdf files without advanced formatting functionality.

However, the app and the current analysis are not without limitations. Because the app does not identify the end user or establish a connection with actual patient medical records, there is no way of knowing whether the observed episodes were utilized for actual patient cases. However, data from New York’s comprehensive ADE reduction quality improvement initiative do suggest that the app is a promising component of a successful intervention campaign to improve anticoagulation safety. In its routine quality improvement role for CMS, IPRO assessed the quarterly rates of bleeding and thromboembolic events resulting in hospitalization within 30 days of an elective surgical procedure among Medicare beneficiaries who had Part D claims for anticoagulants and who were residing within the metro areas of New York State where MAPPP app sessions were identified. In the first calendar quarter of 2016 (ie, prior to app release), IPRO identified 154 ADEs among all eligible cases (154/10,855, 1.42%). In the last quarter of that year (ie, post-MAPPP app launch), among 13,948 cases, 1.13% (157/13,948) ADEs were identified, indicating an approximately 20% relative reduction in the rate of ADEs.

While such improvements cannot be attributed directly to use of the app, efforts are currently underway under a CMS Special Innovation Project awarded to IPRO to facilitate the integration of the app’s logic into the electronic health record systems of 3 health systems as active clinical decision support using Substitutable Medical Applications reusable technologies on Fast Healthcare Interoperability Resources when possible, which will allow a direct assessment of the patient-level impact. Incorporation of the app into existing clinical workflows with executive-level adoption buy-in as facility policy has the potential to affect all patients undergoing relevant procedures and will provide access to the data needed to objectively evaluate the app’s clinical and financial impact. Training on MAPPP app use via recorded webinar and associated patient education materials can be found on the MAPPP app website [25]. Electronic health record integration is anticipated to scale MAPPP use globally. Results of the project are anticipated in 2019.

Similar apps have been developed by reputable and authoritative organizations such as the University of Michigan’s MAQI2 Anticoagulation Toolkit and the ManageAnticoag app developed by the American College of Cardiology [27,28].

### Conclusions

The MAPPP app is a successful means of disseminating current guidance on periprocedural anticoagulant use, as indicated by broad global uptake and upward trends in utilization. Lack of access to provider- and patient-specific data precludes objective evaluation of the clinical impact of the app. An ongoing study incorporating app logic into the electronic health records of 3 health systems will provide a more definitive evaluation of the clinical impact of the app logic.

## Appendix

Multimedia Appendix 1 Management of Anticoagulation in the Peri-Procedural Period Mobile App (MAPPP App). Brief video demonstration of utilizing of the app to guide clinical decisions regarding anticoagulants prior to elective invasive medical procedures and surgeries.

## Abbreviations

ADE: adverse drug event
CMS: Centers for Medicare & Medicaid Services
ED: emergency department
MAPPP: Management of Anticoagulation in the Periprocedural Period
NYSACC: New York State Anticoagulation Coalition
QIN-QIO: Quality Innovation Network-Quality Improvement Organization
